# Nicotinamide adenine dinucleotide induced resistance against root-knot nematode *Meloidogyne hapla* is based on increased tomato basal defense

**DOI:** 10.21307/jofnem-2019-022

**Published:** 2019-04-15

**Authors:** Noor Abdelsamad, H. Regmi, J. Desaeger, P. DiGennaro

**Affiliations:** 1 Department of Entomology and Nematology, College of Agriculture and Animal Science, University of Florida, Gainesville; 2 Gulf Coast Research and Education Center, University of Florida, Wimauma

**Keywords:** Induced resistance, Calcium signaling, *Meloidogyne hapla*, Nicotinamide adenine dinucleotide, Management

## Abstract

Root-knot nematodes (RKN; *Meloidogyne* spp.) are among the most damaging pests to tomato production in the USA and worldwide, with yield losses ranging from 25 to 100%. Host resistance conferred by the *Mi* gene in tomato is effective against some species of RKN (e.g. *M. incognita*, *M. javanica*, and *M. arenaria*); however, there are virulent species and lines including *M. hapla* and *M. eterolobii* that break *Mi*-mediated resistance. Plant innate immunity is another possible form of defense against pathogen attack and is known to be induced by chemical elicitors. Nicotinamide adenine dinucleotide (NAD) is one such chemical elicitor that regulates plant defense responses to multiple biotic stresses. In this study, we investigated the role of NAD in the context of induced tomato innate immunity and RKN pathogenicity in two tomato cultivars; VFN and Rutgers, with and without *Mi*, respectively. Single soil drench application of NAD 24 hr before nematode inoculation significantly induced defense response pathways, reduced infective-juveniles penetration, number of galls, and increased plant mass in both cultivars. Importantly, we observed no direct toxic effects of NAD on nematode viability and infectivity. The results presented here suggest that NAD induces resistance against RKN pathogenicity likely through the accumulation of tomato basal defense responses rather than the direct effect on the infective-juveniles behavior.

Root-knot nematodes (RKNs; *Meloidogyne* spp.) are sedentary endoparasitic nematodes that can infect a wide range of plant species worldwide, which results in approximately $70 billion in crop losses annually (Caboni et al., 2012). *Meloidogyne* spp. is ranked within the top 10 most economically devastating plant-parasitic nematodes, with *Meloidogyne incognita*, *M. arenaria*, *M. hapla*, and *M. javanica* as the four major crop-damaging species (Jones et al., 2013). In tomato, yield loss due to RKNs ranges from 25 to 100%, depending on nematode species, population densities, and tomato cultivar (Seid et al., 2015). Management practices such as chemical nematicides, cover crops, and biological control have been well documented (Monfort et al., 2007; Zasada et al., 2010; Adam et al., 2014). However, factors such as the toxic effect of chemical nematicides on the environment and humans, the wide host range of *Meloidogyne* spp., and the effect of soil properties limit the use of these practices (Pantelelis et al., 2006; Barbary et al., 2015). As a result of these limitations, plant resistance has become the most widely used and effective management approach to control RKN in tomato (Fuller et al., 2008).

In tomato, resistance to RKN is mediated by a single dominant R gene called *Mi-1* that was introduced to cultivated tomato from a single cross with its wild relative *Solanum peruvianum* (formerly *Lycopersicon peruvianum*) (Kaloshian et al., 1998). However, the durability of *Mi* resistance is reduced by sustained high soil temperature (above 28°C), and the emergence of virulent nematode biotypes have been reported likely due to the intensive use of the *Mi* gene that applied selective pressures on nematode populations (Devran et al., 2010). In contrast to genetic resistance, induced resistance is a plant defense mechanism triggered by biological or chemical elicitros, which protects plants against a broad spectrum of biotic stresses including plant-parasitic nematodes. Following application of an elicitor, defense responses like oxidative burst, cell wall fortification, and synthesis of antimicrobial compounds such as pathogenesis-related proteins may be triggered directly or after pathogen attack (Walters et al., 2013). While the mechanisms have yet to be fully understood, elicitors such as acibenzolar-S-methyl (ASM), benzothiadiazole (BTH), DL-β-amino-n-butyric acid (BABA), and salicylic acid (SA) have been reported to induce resistance against RKN (Oka et al., 1999; Chinnasri et al., 2003; Ji et al., 2015) and are likely the result of induced plant resistance.

Plant metabolites are also known to activate defense mechanisms and induce resistance against pathogens (Rojas et al., 2014; Piasecka et al., 2015). Pyridine nucleotides, like nicotinamide adenine dinucleotide (NAD), are important redox carriers and play a crucial signaling role in response to stresses (Hashida et al., 2009). In Arabidopsis, exogenous application of NAD induces the expression of pathogenesis-related (*PR*) genes via the Ca^2+^ dependent signaling pathway, causing accumulation of SA, and enhanced disease resistance to the bacterial pathogen *Pseudomonas syringae* pv. *tomato* (*Pst*) (Zhang and Mou, 2009). Further characterization of NAD-dependent immune responses showed that intracellular NAD-overproducing *nadC* transgenic lines are more resistant to a diverse range of virulent pathogens including *Pst*-*AvrRpm1*, *Dickeya dadantii*, and *Botrytis cinerea* (Pétriacq et al., 2016). NAD can also elicit a defense-related metabolic signature detectable by mass spectrometry that is similar to those triggered by hormones and Pathogen Associated Molecular Pattern (PAMP) such as flagellin (Flg22) and fungal chitin (Pétriacq et al., 2016). The goal of this study was to evaluate NAD-induced plant resistance in a relevant crop and assess its efficacy against RKN.

## Materials and Methods

### Plant materials and growth conditions

Tomato (*Solanum lycopersicum*) lines used in this work included *S. lycopersicum* cv. Rutgers which does not have the *Mi-1* gene, and cv. VFN (resistance to Verticillium, Fusarium, and root-knot Nematode) with the *Mi-1* gene (Urban farmer LLC, Westfield, IN). Both cultivars are susceptible to *M. hapla* infection. Seeds were germinated in a mixture composed of sand and soil in a 1:1 (v/v) ration. Plants were grown in a growth chamber at 24°C and a 16-h light and 8-h dark regime, with daily watering. After two weeks, seedlings were washed and transferred to trays containing sand mix and kept on the bench for one week to recover before nematode inoculations.

### Nematode viability

To test the negative effect of NAD on nematode mortality and infectivity, 400 J2 of *M. hapla* were incubated in six-well plates, each well contained 2 ml of 5 mM NAD solution or water as control under dark conditions at room temperature for 2 d. Live and dead nematodes were counted under a dissecting microscope and the percentage of dead nematodes was counted. Each treatment included six technical replicates, and the experiment was repeated three times. To investigate the effect of NAD on nematode infectivity, juveniles were collected after 2 d incubation in NAD or water, washed three times in water, and inoculated onto universal susceptible Rutgers tomato plants at100 J2 per plant.

### Nematode inoculation

The root-knot nematode, *M. hapla*, culture was maintained on the susceptible *S. lycopersicum* cv. Rutgers under greenhouse temperature and light conditions. Nematode eggs were extracted from infected tomato roots using 10% commercial bleach and 40% sucrose solution (Hussey and Barker, 1973), eggs were then incubated at 25°C in hatching bowls, second-stage juveniles (J2s) were collected after 5 d. Pretreatment with NAD was performed as follows: 20 d old seedlings of cvs. Rutgers and VFN were soil drenched with 10 ml of 5 mM NAD solution (or water control), and 1 d later they were inoculated with approximately 350 to 400 J2 of *M. hapla* per plant by distributing the nematode suspension into three holes (2 cm deep) in the soil.

To study the effect of NAD on *M. hapla* development in tomato plants, two cultivars (VFN, and Rutgers), two chemical treatments (NAD and water), two sampling time points at 2, and 15 dpi (days post-inoculation), and two nematode inoculation levels (inoculated and non-inoculated) were arranged in a completely randomized design. There were five replicate pots per treatment combination and the experiment was repeated three times. Five plants were destructively sampled at 2 and 15 dpi and used to quantify J2 penetration, and number of galls, respectively. To visualize the nematodes inside the roots, tomato seedlings were washed to remove sand particles and the roots were placed in 10% commercial bleach solution for 4 min, then incubated in tap water for at least 15 min to remove excess bleach. Roots were then boiled in 3.5% acid fusion stain, after which they were washed with tap water and were boiled briefly in acidified glycerol (10:100 HCl/glycerol, vol/vol) for destaining. Number of J2 and galls were counted using stereomicroscope (LeicaMz6, Leica Microsystems, IL, USA). Fresh root and shoot weight were measured at 15 dpi on five plants per treatment, by rinsing in tap water to remove sand particles, and drying the excess water using paper towels. There were three independent biological experiments, each experiment has five biological replicates per treatment.

### RNA extraction, cDNA synthesis, and real-time PCR

For RNA extraction, whole tomato roots were collected 24 h after NAD application then grounded to a fine powder in liquid nitrogen, and total RNA was extracted using Trizol reagent (Invitrogen, Carlsbad, CA, USA). DNA contamination was removed using RNase-free DNase I (Invitrogen) following the manufacturer’s instructions. RNA was quantified using a Nano-Drop 1,000 spectrophotometer (Thermo Scientific, Wilmington, DE), and integrity was verified on 1% agarose gel. First-strand cDNA was synthesized from 0.5 µg of total RNA using iScript cDNA synthesis kit (Bio-Rad, Hercules, CA, USA).

Quantitative PCR was performed using iTaq universal SYBR Green Supermix (Bio-Rad) and Applied Biosystems Step One Plus detection system. The reaction mix consisted of 5 µl master mix, 0.5 µl of reverse and forward primers (500 nM final concentration), 2 µl of diluted cDNA (10 ng final concentration), and the final volume was adjusted to 10 µl with RNase DNase free water (Invitrogen). The primers sequences used for real-time PCR are listed in Table 1. The thermal cycling protocol was 2 min at 95°C, 40 cycles of 3 s at 95°C, 30 s at 60°C, followed by melting curve data collection to check for nonspecific amplification and primer dimers. Relative gene expression was calculated using the 2^−ΔΔct^ method (Livak et al., 2001), in which the transcription levels of the target genes in control seedlings (J2 inoculated and water treated) were used as reference for expression analysis, and Ubiquitin was used as the internal control gene expression.

**Table 1:** Primers used in this study for qRT-PCR.

Gene	Description	Forward primer (5′-3′)	Reverse primer (5′-3′)	Reference
*SlPR1*	Lycopersicon esculentum PR1a	CCAAGACTATCTTGCGGTTCA	CGCTCTTGAGTTGGCATAGT	Li et al. (2015a)
*SlPR2*	beta-1.3-glucanase	TCCAGGTAGAGACAGTGGTAAA	CCTAAATATGTCGCGGTTGAGA	Li et al. (2015a)
*SlPR5*	Lycopersicon esculentum PR5	CCCAAACACCCTAGCTGAAT	GGGCGAAAGTCATCGGTATATTA	Li et al. (2015a)
*SlPAL*	Phenylalanine ammonia-lyase	TGATGAACGGAAAGCCTGAA	CTGAGCTGCCTTGACATAAGA	Li et al. (2015a)
*CDPK15*	Calcium-dependent protein kinase	ACGGACAATAGTGGGACA	TGCTTAACTTCAGCCTCC	Hu et al. (2016)
*RbohB*	Respiratory burst oxidase homologs	AGGGAATGATAGAGCGTCG	CATCGTCATTGGACTTGGC	Li et al. (2015b)
*Ubi3*	Ubiquitin	GTGTGGGCTCACCTACGTTT	ACAATCCCAAGGGTTGTCAC	Bhattarai et al. (2008)

### Calcium-sensitive dye labeling

Tomato roots from both cultivars were drenched with NAD 5 mM solution for 24 hr then collected and rinsed in tap water to remove sand particles. Roots were then dissected into small sections (2-3 cm) to represent the whole root system. Sections were incubated in six-well plates containing 15 µM Calcium Green-1 (Life Technologies), which fluoresces after binding to Ca^2+^. The plates were incubated for 1 hr at room temperature under dark conditions. As a control, tomato roots were treated with water following the same experimental conditions. Calcium Green-1 was excited at 506 nm and the emission detected at 530 nm (Carl Zeiss AXI0).

### Statistical analysis

Statistical analysis was performed using procedures in SAS 9.4 software (SAS Inst. Inc., Cary, NC, USA). Raw data were checked for normality and homogeneity of variance using (Quantile–quantile plot, Shapiro–Wilks, Anderson–Darling, Cramer–von Mises, and Levene’s tests). Data were pooled following testing for homogeneity of variance. For comparison between NAD and water treatments, changes in number of juveniles, galls, shoot fresh weight, and relative gene expression levels were analyzed using Student’s *t-*test (*P* < 0.05).

## Results

### NAD has no direct effect on RKN viability

**Figure 1:**
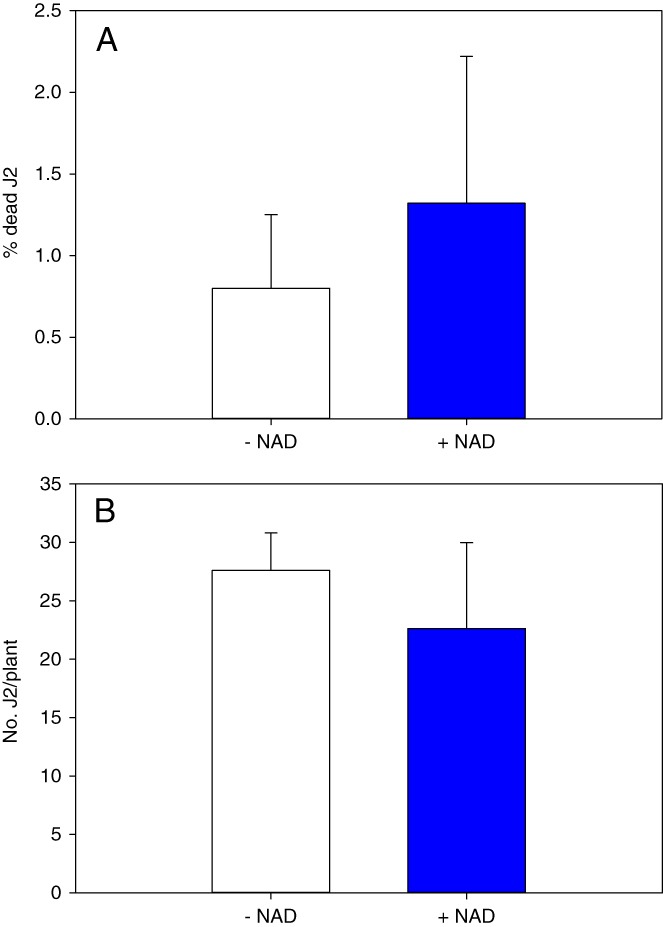
Effect of Nicotinamide adenine dinucleotide (NAD) treatment on *Meloidogyne hapla* viability and infectivity. (A) Percentage of dead J2 after incubation for 48 hr in NAD or water (control). Bars represent the average mean of 18 biological samples ± standard deviation. (B) Infectivity of NAD-incubated and water-incubated *M. hapla* juveniles in tomato roots 48 hr post-inoculation. Bars represent the average mean of fourteen biological samples ± standard deviation. Statistical significance of the difference was tested using Student’s *t*-test (*=*P* < 0.05).

To examine whether NAD has a direct impact on J2 survival, nematodes were incubated in 5 mM NAD solution or water for 48 hr then live J2 were counted (straight in shape juveniles were considered dead). There were three independent biological runs, each run has six biological replicates per each treatment. The percentage of dead nematodes in NAD treated J2 (1.32% ± 0.9%) was not significantly different from that in water treated J2 (0.8% ± 0.45%) (Fig. 1A), indicating that NAD has no direct nematicidal effect on *M. hapla* J2 survival. To further investigate the effect of NAD on root-knot nematode behavior, NAD and water treated infective juveniles were inoculated on universal susceptible Rutgers tomato cultivar. There were four independent biological experiments, the first and second experiment each has two biological replicates per treatment, third and fourth experiment each has five biological replicates per treatment. At 48 hr post-inoculation (hpi), no significant difference was observed between the numbers of J2 that penetrated tomato roots in NAD treated nematodes (45.07 ± 25.99) compared to water treated control (41.28 ± 24.26) (Fig. 1B). Collectively, these data indicate that NAD 5 mM solution has no toxic effect on RKN viability and infectivity.

### NAD induces plant resistance against RKN in tomato

**Figure 2:**
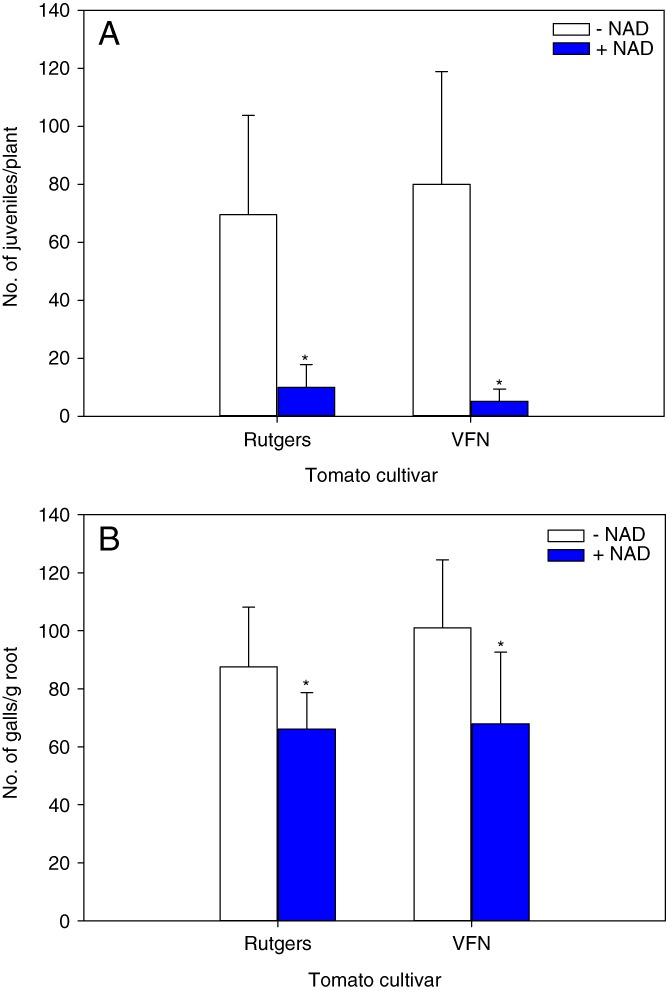
Effect of Nicotinamide adenine dinucleotide (NAD) on the development of *Meloidogyne hapla* in tomato plants. Tomato seedlings (20-d-old) were soil drenched with 5 mM NAD solution or water (−NAD) one day before *M. hapla* inoculation. (A) Number of J2 inside tomato cultivars; Rutgers (*Mi−*) and VFN (*Mi+*) 48 hr post-inoculation (hpi). (B) The number of galls per gram of root in tomato cultivars Rutgers and VFN 15 d post-inoculation (dpi). Bars represent the average mean of 15 biological samples ± standard deviation. Statistical significance of the difference was tested using Student’s *t*-test (*=*P* < 0.05).

A previous study by Zhang and Mou (2009) showed that 5 mM NAD is sufficient to induce pathogenesis-related proteins (*PR*) and enhance Arabidopsis resistance against bacterial pathogen *Pseudomonas syringae*. To assess whether NAD exogenous application could induce resistance against RKN in tomato, we analyzed juveniles penetration and gall formation in tomato cultivars with and without nematode resistance *Mi* gene. There were three independent biological experiments, each experiment has five biological replicates per treatment. At 48 hpi, NAD treated plants had significantly reduced nematode penetration by 85.6 and 93.5% in Rutgers (*Mi−*) and VFN (*Mi+*), respectively (Fig. 2A). To evaluate whether this one-time NAD application, 24 hr prior to inoculation with RKN, is sufficient to reduce nematode development, we measured galling per gram of root at 15 dpi. Compared to water inoculated control, NAD pretreatment reduced number of galls by 25 and 32% in Rutgers and VFN plants, respectively (Fig. 2B). However, no significant difference in number of penetrating juveniles or galling was observed between cultivars within the same treatment (water or NAD), suggesting that NAD-induced resistance in tomato against RKN is *Mi* gene-independent (Fig. 2A,B).

### NAD application has a positive effect on tomato growth

Induced resistance in plants is usually associated with fitness costs (Huot et al., 2014). To examine the effect of NAD on tomato growth, we measured above-ground (shoot and leaves) fresh plant weight treated with water or 5 mM NAD 15 d after germination in the presence or absence of nematode infection. There were three independent biological experiments, each experiment has five biological replicates per treatment. In both cultivars, a significant increase in plant biomass was observed in response to NAD application compared to water treated control. However, no significant difference was detected between nematode infested and non-infested plants in either cultivar (Table 2).

**Table 2:** Effect of NAD application on shoot fresh weight (g) of susceptible and resistance tomato cultivars in the presence or absence of *Meloidogyne hapla* ^a^.

	Inoculated		Non-inoculated	
	Cultivar^b^			
Treatment^c^	Rutgers	VFN	Rutgers	VFN
Control	1.12 (0.39)	0.93 (0.23)	0.91 (0.22)	0.86 (0.43)
NAD	1.98 (0.35)*	2.17 (0.30)*	1.75 (0.56)*	1.85 (0.70)*

**Notes:** ^a^Values represent the mean (standard deviation in parentheses) shoot fresh weight (stem and leaves) of 15 plants. Asterisk within the same column indicates the significant difference (*P*<0.05) between nicotinamide adenine dinucleotide (NAD) and control; ^b^root-knot nematode susceptible cultivar Rutgers (*Mi*−) and RKN resistant cultivar VFN (*Mi*+) were inoculated with 300 to 400 *M. hapla* J2 or water for the non-inoculated plants; ^c^21 d-old plants were soil drenched with 5 mM nicotinamide adenine dinucleotide (NAD) solution or water for control 24 hr before J2 inoculation, samples were collected 15 d after nicotinamide adenine dinucleotide (NAD) treatment.

### NAD triggers defense gene expression in tomato roots

**Figure 3:**
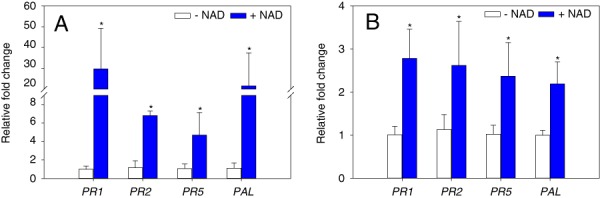
Relative expression of defense-related genes in response to NAD treatment. 20-d-old plants were treated with 5 mM NAD, or water (−NAD) and roots were harvested 24 hr post application. Expression levels of indicated genes; pathogenesis-related protein 1a (*PR1*), beta-1,3-glucanase (*PR2*), pathogenesis-related protein 5× (*PR5*), and phenylalanine ammonia-lyase (*PAL*) were quantified in tomato cultivars (A) Rutgers (*Mi−*), and (B) VFN (*Mi+*) using qRT-PCR. Ubiquitin gene (*Ubi3*) was used as an internal control. Gene expression values are presented relative to water treated control (−NAD). Bars represent the average mean of six biological samples ± standard deviation. Statistical significance of the difference was tested using Student’s *t*-test (*=*P* < 0.05).

Plant resistance is often measured by the induction of defense-related gene expression (Van Loon et al., 2006). The expression levels of genes encoding pathogenesis-related proteins (*PR1*, *PR2*, and *PR5*) and phenylalanine ammonia-lyase (*PAL*) were examined in tomato roots treated with NAD or water for 24 hr using RT-qPCR. There were two independent biological runs, each run has three biological replicates per each treatment. In both tomato cultivars, the expression levels of all *PR* genes tested were significantly up-regulated in response to NAD application compared to water treated control (Fig. 3A,B). *PAL* is a key enzyme in the phenylalanine pathway and also involved in resistance against plant pathogens (Huang et al., 2010). The expression level of *PAL* was significantly up-regulated in NAD treated seedlings compared to water treated ones (Fig. 3A,B).

### NAD increased calcium accumulation and expression of calcium-response genes in roots

**Figure 4:**
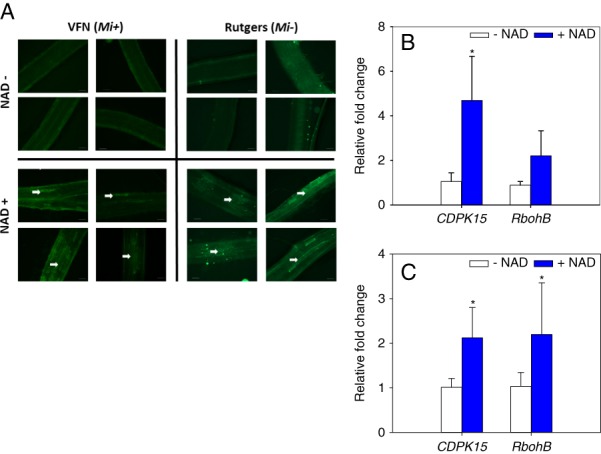
Nicotinamide adenine dinucleotide (NAD) treatment induces calcium signaling. (A) Microscopic observation of Ca^2+^ levels in roots of tomato cultivars Rutgers and VFN. Tomato seedlings were treated with 10 ml 5 mM NAD or control (−NAD) for 24 hr, then root sections (2 cm) were labeled with Ca^2+^ – sensitive dye, Calcium Green-1. Note Ca^2+^ accumulation as revealed by green fluorescence microscopy (white arrows). Scale bars = 200 μm. (B) and (C) Gene expression analysis of calcium-dependent protein kinase (*CDPK15*) and respiratory burst oxidase homologs (*RbohB*) in tomato cultivars Rutgers and VFN, respectively. Real-time PCR analysis was performed to determine the transcript levels of *CDPK15* and *RbohB* in tomato roots pretreated with 5 mM NAD or control (−NAD) for 24 hr. Bars represent the average mean of six biological samples ± standard deviation. Statistical significance of the difference was tested using Student’s *t*-test (*=*P* < 0.05).

Accumulation of intracellular and/or extracellular calcium ions is an early signaling event in response to pathogen attack (Ranty et al., 2016) and there is a link between Ca^2+^ signaling and NAD-mediated resistance (Zhang and Mou, 2009). To further investigate the mechanisms of NAD-induced resistance against RKN infection, calcium ions in pretreated NAD or water roots were labeled with Calcium Green-1 dye and visualized under a fluorescent microscope. In both cultivars, a strong fluorescence in root epidermal cells of sections pretreated with NAD for 24 hr was observed, whereas relatively little fluorescence was detected in water treated root sections (Fig. 4). In total, 10 root sections treated with NAD or water were visualized and showed a similar pattern of green fluorescence. In addition to microscopic observation, we also quantified the transcript level of calcium-dependent protein kinase (*CDPK15*) and respiratory burst oxidase homolog (*RbohB*) in tomato seedlings pretreated with NAD or water for 24 hr using qRT-PCR. There were two independent biological experiments, each experiment has three biological replicates per treatment. In both cultivars, *CDPK15* gene expression was significantly up-regulated in roots treated with NAD in comparison to roots treated with water (Fig. 4B), whereas *RbohB* gene expression was only significantly up-regulated in roots of VFN (*Mi*+) cultivar treated with NAD compared to control (Fig. 4C).

## Discussion

Pyridine nucleotides, including NAD, are key regulators of cellular oxidation reactions and serves as a cofactor for important enzymes involved in primary and secondary metabolisms (Pétriacq et al., 2013). In addition to this role, NAD and its derivatives induce plant defense responses as they participate in cellular signaling mechanisms related to pathogen infection in Arabidopsis (Zhang and Mou, 2009; Pétriacq et al., 2016). However, the role of NAD in resistance against plant-parasitic nematodes remains largely unknown. Here, we present a new role for NAD in resistance signaling in tomato against the root-knot nematode (RKN; *M. hapla*) and provide a characterization of multiple associated defense mechanisms.

NAD-induced resistance to biotic stresses is well established in Arabidopsis. For example, infiltration of Arabidopsis seedlings with NAD enhances resistance to the bacterial pathogen *Pseudomonas syringae* (Zhang and Mou, 2009). Using Arabidopsis lines that accumulate NAD upon treatment with quinolinic acid, Pétriacq et al. (2016) found that transient increase in NAD pools induced Arabidopsis resistance to pathogens like *Pseudomonas syringae, Dickeya dadantii,* and *Botrytis cinerea*. Furthermore, constitutive expression of *NMNAT* gene, which encodes an important enzyme in NAD biosynthesis pathway, resulted in enhanced disease resistance to *Fusarium graminearum* in Arabidopsis (Miwa et al., 2017).

We showed that NAD soil drench application for 24 hr before nematodes inoculation made tomato roots more difficult for *M. hapla* to penetrate, as indicated by a lower number of juveniles in roots at 48 hpi. In addition, gall formation was reduced by 25 to 30% in NAD treated plants compared to control, suggesting an inhibitory effect on the development of *M. hapla* juveniles inside tomato roots. Importantly, no direct toxic effect on RKN mortality or infectivity was observed on juveniles incubated in NAD solution for 48 hr. At the molecular level, soil drench application of 5 mM NAD solution enhances the expression of defense-related genes such as pathogenesis-related proteins and genes involved in calcium signaling. Taken together, these results suggest that the enhanced resistance in NAD treated plants is likely through the accumulation of tomato basal defense responses, rather than a direct toxic effect on infective-juvenile behavior.

To define the possible mechanisms that contribute to NAD-induced resistance against RKN, we examined the expression levels of genes encoding pathogenesis-related proteins such as *PR1, PR2, PR5*, and phenylalanine ammonia-lyase (*PAL*) gene, which are commonly considered as marker genes for systemic acquired resistance (SAR) (Molinari et al., 2014). Pharmacological and genetic analyses have demonstrated that NAD induces salicylic acid accumulation and *PR* genes expression (Pétriacq et al., 2012). As described by Zhang and Mou (2009), exogenous NAD application enhances Arabidopsis resistance to *Pseudomonas syringae* via the expression of *PR* genes. Arabidopsis leaves pretreated with an NAD precursor nicotinamide mononucleotide (NMN) induce *PR1* gene expression and reduce *Fusarium graminearum* abundance (Miwa et al., 2017). Similarly, our results showed that NAD soil drench for 24 hr is sufficient to induce the expression of *PR1, PR2, PR5*, and *PAL* genes in tomato as well as reduce RKN infection.

Calcium signaling is a central biochemical response in plant defense (Ranty et al., 2016). Chelation of Ca^2+^ by EGTA inhibits NAD-mediated *PR* genes expression, suggesting the importance of calcium signaling in NAD-mediated resistance (Zhang and Mou, 2009). In this study, microscopy observations showed treatment with NAD resulted in the accumulation of calcium levels in tomato roots, highlighting the importance of Ca^2+^ signaling in NAD-mediated resistance against RKN. This is similar to the response in potato, where microscopic observation and transgenic analysis showed that Ca^2+^ plays a role in the *R*_*Mc1(blb)*_-mediated resistance against *Meloidogyne chitwoodi* (Davies et al., 2015). The calcium signaling pathway could also interact with other cellular signaling systems like reactive oxygen species (ROS). In this study, we did not measure ROS levels but we quantified by the expression of respiratory burst oxidase homolog (*RBOH*). Increases in intracellular Ca^2+^ is perceived by calcium sensor-effector known as calcium-dependent protein kinases (CDPKs), which phosphorylates *RBOH* and subsequently lead to the release of *Rboh*-mediated reactive oxygen species (ROS) production (Zhang et al., 2014). In line with our microscopic observation, the expression of both *CDPK15* and *RbohB* was significantly enhanced in NAD treated roots with the noted exception of *RbohB* in Rutgers cultivar, the reason for this exception is unknown; however, one possible explanation could be that the NAD-induced calcium signaling is not enough to enhance *RbohB* expression in Rutgers cultivar. Taken together, these data are suggesting that NAD application for 24 hr before nematode infection can induce Ca^2+^ signaling and subsequently enhance tomato resistance against RKN.

It is well established that constitutive or induced plant resistance is generally associated with growth-defense trade-offs (Huot et al., 2014). For example, under field conditions, nematode reproduction was significantly lower on the RKN resistant cultivar Motelle (*Mi-1.2+*) than on the susceptible cultivar Moneymaker (*Mi-1.2−*), however, no significant differences in total yield of mature fruits or dry root weight were observed between the two cultivars (Corbett et al., 2011). Similarly, foliar application of plant defense activators such as jasmonic acid and its derivatives successfully control nematode penetration and development in potato; however, plants treated with *cis*-jasmone did not produce tubers and showed phytotoxicity symptoms (Vieira dos Santos et al., 2013). We suspected a similar phenotype may be present in the presence of exogenous NAD. However, NAD treated seedlings showed an increase in above-ground biomass in the presence or absence of nematode infection. This positive effect could be due to the importance of NAD in the plant growth and developmental process (Hashida et al., 2009).

Here, we present evidence that exogenous NAD application can induce tomato resistance against RKN, as indicated by a lower number of nematodes and galls, enhanced expression of defense-related genes, and accumulation of calcium levels in NAD treated roots compared to control. However, no difference in the number of nematodes or galls was observed between cultivars of NAD treatment, suggesting that NAD-induced resistance is independent of the *Mi* gene-mediated resistance. This study highlights the importance of primary metabolites as a potential inducer of resistance against RKN. In Arabidopsis, a lectin receptor kinase has been reported as a first potential eNAD^+^ receptor, which is also required for resistance against *Pseudomonas syringae* (Wang et al., 2017). Interestingly, Shidore et al. (2017) showed that *Xanthomonas* type III effector (*AvrRxo1*) can phosphorylate NAD *in planta*, which is a new strategy by bacterial pathogens to target central metabolite and subsequently manipulate the host cells to its own. We expect future work will focus on functionally characterizing NAD signaling components to understand their role in plant-RKN interaction.
